# Ergonomics, Health, and Perceptions about Remote Domestic Workposts: Study in Areas of City of João Pessoa, Paraíba, Brazil

**DOI:** 10.3390/ijerph21070941

**Published:** 2024-07-19

**Authors:** Luiz Bueno Silva, Carmem Julianne Beserra Melo, Adriana Gomes Lisboa de Souza, Lucas Guedes de Oliveira

**Affiliations:** Department of Production Engineering, Federal University of Paraíba, João Pessoa 58051-970, Brazil; carmem_julianne@hotmail.com (C.J.B.M.); anairdasouza@yahoo.com.br (A.G.L.d.S.); lucasguedesdeoliveira@gmail.com (L.G.d.O.)

**Keywords:** home office, remote home workstation, ergonomics, health, lighting, temperature, non-ionizing radiation

## Abstract

Home office (HO) stands out as one of the most promising and popular forms of teleworking, especially after the COVID-19 pandemic. Therefore, many companies want to implement or maintain this working method, given its numerous advantages. However, there are adverse effects that are mainly related to physical and mental health. This article presents ergonomic analyses of HOs in neighborhoods considered heat islands. Temperature levels, extreme low-frequency non-ionizing radiation (ELF-NIR), illuminance, physical layout characteristics, and physiological parameters of teleworkers were measured. The results reveal that 92% of these professionals work 6 to 8 h daily with an ambient temperature between 25 and 30 °C, illumination levels in the range 11.20–290 Lux, and ELF-NIR > 0.4 µT. The majority of teleworkers are overweight (BMI > 24.9), and some of them have blood pressure higher than average values (129 mmHg for systolic and 84 mmHg for diastolic) in addition to a reduction in the number of red blood cells and hematocrits. Symptoms such as burning sensation, dryness, tired eyes, redness, itching, and photophobia (light sensitivity) show a 68.95% similarity. These HOs do not meet the required ergonomic and health standards.

## 1. Introduction

Remote work is a form of paid employment conducted away from the company’s headquarters and requires the use of technology for communication between the employee and the organization [1]. The home office (HO) stands out among its variations, having gained significant visibility during COVID-19 as a work and educational strategy facilitating social isolation imposed by the pandemic. The adoption of the HO has been very intense in recent years, especially during the pandemic, leading to an increase from 15% to over 60% of the United States population working from home [2]. Similarly, in Canada, there was an increase from 4% to 32% of Canadians working from home [3]. In Australia, between 2013 and 2020, the Australian Public Service saw an increase in remote work from 10% to 57% of its employees [4]. As of November 2020, approximately 7.33 million employees in Brazil were working remotely, representing 9.6% of the total workforce [5]. From a post-pandemic perspective, many companies and universities have adopted fully remote or hybrid work models, citing benefits such as cost savings on physical space, global hiring opportunities, flexible hours, autonomous work processes, better work-life balance, and improved urban quality of life through reduced traffic, lower pollution, and less strain on public transportation [6,7,8].

In its characteristics, the home office (HO) is a form of work conducted within the teleworker’s own residence, with a workstation implemented using personal resources situated near household appliances in a building structured solely for residential purposes. In this work environment, which is not easily accessible to regulatory agencies, applying ergonomic principles becomes essential to ensure the teleworker’s health, well-being, and productivity. Some studies indicate the need for research to explore the real impact of remote work on various aspects of the lives of professionals, students, and educators [9]. The main issues identified include the reduction in interpersonal relationships at work, difficulties in managing the work-family interface, changes in workload and work rhythms, lack of ergonomic furniture at the workstation, and the absence of regulatory standards that define the work format and specify parameters for the physical environment [10,11]. Regarding HO furniture, the review by [12] highlights the lack of ergonomic conditions at the HO workstation, which can cause musculoskeletal injuries, sleep disorders, and vision problems [8]. Additionally, sedentary behavior and social isolation, driven by long hours at the computer, contribute to the development or exacerbation of other physical illnesses, stress, and psychosocial problems [13].

In addition to ergonomic issues related to furniture, other risks inherent to home and office environments can be found in the office environment, which can influence the health, well-being, and productivity of teleworkers. Among the main concerns associated with the internal environment of offices is the quality of the internal environment, which encompasses the parameters of thermal comfort (TC), lighting levels, noise levels, and air quality [14,15,16,17]. In residential environments, the quality of the internal environment, as perceived by users, is mainly associated with issues of temperature, lighting, and the layout of the environment [18]. Another health concern in residential environments inherent in modern life is the issue of exposure to electromagnetic fields due to the indiscriminate use of many indoor devices capable of emitting non-ionizing radiation of extremely low frequency (ELF-NIR) and radiofrequency (RF-NIR) [19].

This study analyzed the variables of thermal comfort (TC), lighting, and ELF-NIR because TC is a parameter of great importance in the office environment as it directly affects the well-being and productivity of the worker [20]. Lighting conditions also have a strong impact on eye health, which can cause fatigue and reduce the quality of sleep, and have a corresponding impact on productivity [21]. NIR is associated with metabolic changes and cancer risk and can also cause changes in brain wave patterns during sleep and non-specific symptoms during and after activity, such as headaches, nausea, and dizziness in people who are so-called electrosensitive [22,23,24,25]. The need to use electronic equipment such as computers, wi-fi, cell phones, and tablets for several hours in a residential environment with other household appliances raises many questions about the possible health effects associated with exposure to ELF-NIR [26].

In this context, the study of ergonomics, health, and perception regarding remote home workstations is crucial in improving working conditions by identifying health risks and enhancing the productivity of teleworkers in a home office setting. The purpose of this article is to present an assessment of remote home workstations in vertical residential buildings located in heat island regions of João Pessoa, while considering ergonomic factors, teleworkers’ perceptions, lighting, thermal comfort, non-ionizing radiation, and health parameters.

## 2. Materials and Methods

Ergonomic analysis of the home office requires a comprehensive view of the work environment, taking into account various internal aspects as well as the specifics of the environment. The most relevant variables in home offices are associated with chair adjustment, positioning of the workstation in relation to the window, and posture, which lead to health problems such as eyestrain and pain in the arms, shoulders, and back [27]. However, physical characteristics such as the room, the size of the area, the height of the ceiling, geographical orientation, and shading devices must be taken into account, as the study by [28] emphasizes that ergonomic factors and characteristics of remote work, such as the work environment and furniture, are associated with musculoskeletal disorders.

Having a dedicated room in one’s home to work from increases productivity [29]. The size of the office area influences the quality and efficiency of work [30] and is strongly related to acoustics, privacy, and satisfaction with temperature, ventilation, and lighting [31]. Ceiling height is a determining factor in the positioning of climate control devices [32] and helps to provide a thermally uniform environment [33]. The geographical orientation of a room can significantly influence temperature variation [34]. The use of shading devices improves thermal comfort, reduces energy consumption [35], contributes to a better visual environment by evenly distributing natural light [36], and minimizes the risk of glare [37].

This article results from a study initially submitted to the Health Ethics Committee, located at the Health Sciences Center of the Federal University of Paraíba, Brazilian Platform, for the approval of the scientific design, methodology, and procedures of the study, with the favorable research opinion number 6.079.720. The sample consisted of 13 professionals (convenience sample) from various fields recruited through an online survey. The inclusion criteria were workers (female or male) aged over 20 years, with employment and remuneration, who performed remote activities during the pandemic or who started working in a home office or hybrid model after the pandemic period for at least 3 consecutive days a week, 6–8 h a day. The measurements were carried out in remote residential environments only in vertical buildings up to the third floor, located in heat islands in João Pessoa. The neighborhoods with considerable heat islands were identified according to the Urban Climate Map of João Pessoa [38]. The internal environment also needed to have a workstation with natural ventilation.

### 2.1. Perception of the Professionals

The VERAM (Visual Ergonomic Risk Assessment) method was employed to evaluate the professionals’ perceptions. This method consists of an online questionnaire regarding ergonomic risks, including variables such as fatigue, lighting conditions, and musculoskeletal discomfort [39]. The method encompasses four stages: (a) Questionnaire for the professional; (b) Technical measurements and subjective evaluations; (c) Follow-up questions based on the professional’s responses; (d) Recommendations section. A form was created on the Google Forms platform to gather the responses, and the link was sent to the participants via WhatsApp.

### 2.2. Thermal Comfort

For the study on thermal comfort, a TGD400 thermal stress meter, calibrated by INSTRUTHERM/SP Certificate 149003R/23 [40], was utilized. The meter was installed near the HO workstation, perpendicular to the airflow, at abdomen height from the ground, in accordance with the provisions of [40], for the entire duration of the telework shift. Regarding the perception of thermal comfort, a questionnaire was administered containing questions about the students’ perceptions and evaluations based on 7-point scales of thermal perception and preferences as specified by [41].

### 2.3. Lighting

Lighting in office environments is one of the most important parameters and can have an impact on workers’ health and productivity [42]. For teleworkers, this is a source of great discomfort when carrying out work tasks in work environments [43].

A digital lux meter with a data logger, Akron model KR852, was used for the lighting measurements at the HO workstation. This device features a photocell corrected for human eye sensitivity and incidence angle, properly calibrated, and positioned on the horizontal visual task plane [44,45]. The illumination levels measured in lux were recorded throughout the workday, with 12 measurements per minute.

### 2.4. Evaluation of Non-Ionising Radiation Levels

With technological evolution, environments are being affected by electromagnetic pollution [46], alerting us to the need to study non-ionizing radiation. The possible adverse effects, such as increased free radicals in cells and reduced cognitive function, make non-ionizing radiation a relevant variable in the home office environment [47].

Magnetic field data were obtained using an Aaronia USA Spectrum Analyser, model Spectran NF-5035, operating in the 1 to 120 Hz range, positioned near the teleworker during work activities. For the analysis of ELF-NIR data, we considered only values in the 60 Hz frequency range, corresponding to Brazil’s power grid frequency.

### 2.5. Analysis of Physiological Parameters

Several physiological parameters were assessed, including blood pressure, heart rate, cortisol levels in blood and saliva (morning and afternoon), and blood cell quality. Blood pressure and heart rate were measured throughout the workday at 15-min intervals using the Contec ABPM50 24H Ambulatory Blood Pressure Monitor (Contec Medical Systems Co., Ltd., Qinhuangdao, China), installed on the left arm, following the recommendations outlined in the 6th Guidelines for Ambulatory Blood Pressure Monitoring and the 4th Guidelines for Home Blood Pressure Monitoring [48] Oxygen (O_2_) levels were measured using a G-tech LED Oled Graph oximeter (Beijing Choice Electronic Technology Co., Ltd., Beijing, China), in contact with the fingertip, following the recommendations of the Pulse Oximetry Monitoring in Primary Health Care Advisory (APS), with measurements taken at the start and end of the work shift [49]. Stress levels were assessed through cortisol hormone levels detected in saliva (2 samples per day) using a Salivette device (Prismalab Comércio de Produtos Laboratoriais LTDA, Rio de Janeiro, Brazil). Blood samples (1 per day) were collected by a qualified nurse, with 10 mL taken (5 mL for blood cell quality testing and 5 mL for cortisol level testing) and sent for analysis at a biochemical laboratory. The number and characteristics of lymphocytes in the blood were investigated via automated counting and optical microscopy analysis, both conducted at the biochemical analysis laboratory. Finally, a Smart Band MI BAND pedometer watch (Xiaomi, Beijing, China), worn on the wrist at the start of the workday and removed at the end, recorded the number of steps taken during work activities.

### 2.6. Statistical Analysis

To analyze comfort and health data, descriptive statistics of central tendency measures were utilized. Parametric and non-parametric tests were employed for correlational and comparative studies, and multivariate analysis through clustering and Principal Component Analysis was conducted to evaluate the similarities between perceptual and ergonomic variables.

For this, the mean and standard deviation of illumination, temperature, and non-ionizing radiation values were extracted. Then, multivariate analysis was carried out to treat the professionals’ perceptions. Initially, cluster analysis was performed, followed by principal component analysis. These statistical techniques transform the original set of variables into a substantially smaller set, facilitating data comprehension [50].

Cluster analysis is a multivariate technique that aims to separate elements into groups such that the elements within the same group are homogeneous and elements from different groups are heterogeneous [51,52]. These elements are grouped according to their similarity.

Principal Component Analysis transforms a set of original variables into another set of latent variables called principal components. Each principal component is a combination of the original variables but is linearly independent. Its primary objective is to retain the maximum amount of information from the total variation in the data [53].

## 3. Results

### 3.1. Sociodemographic and Health Profile

Sociodemographic characteristics, health information, and physiological parameters were collected through questionnaires developed for this research and the volunteers’ measurements, which aimed to outline the sample profile and identify pre-existing morbidities.

Table 1 shows that the group of volunteers (VOL) consists of men (46%) and women (54%), with an average age of 41.85 ± 9.5 years, mostly married, and 54% without children. Nearly all teleworkers (92%) have higher education degrees, working in either the public (46%) or private (54%) sectors. They have been engaged in telework for over one year, working 8 to 6 h per day, 5 days a week.

### 3.2. Health Profile

Table 2 indicates that 8 out of the 13 volunteers are overweight (BMI > 24.9 and waist circumference > 88 cm for women), according to the guidelines of the Brazilian Society of Obesity [54]. None of these teleworkers are smokers, though some consume alcohol sporadically. They exhibit sedentary behavior, reporting no regular physical activity and recording a low number of steps during their workday, as measured by a pedometer watch, falling below the 10,000 steps recommended by the WHO for the prevention of cardiovascular diseases, cancer, and type 2 diabetes [55]

Despite the long hours spent working in front of a computer, the teleworkers report good overall health, experiencing occasional body pains, primarily in the head, eyes, and neck regions. Additional complaints include reflux, nausea, poor circulation, tingling, forgetfulness, and anxiety.

**Figure 1:**
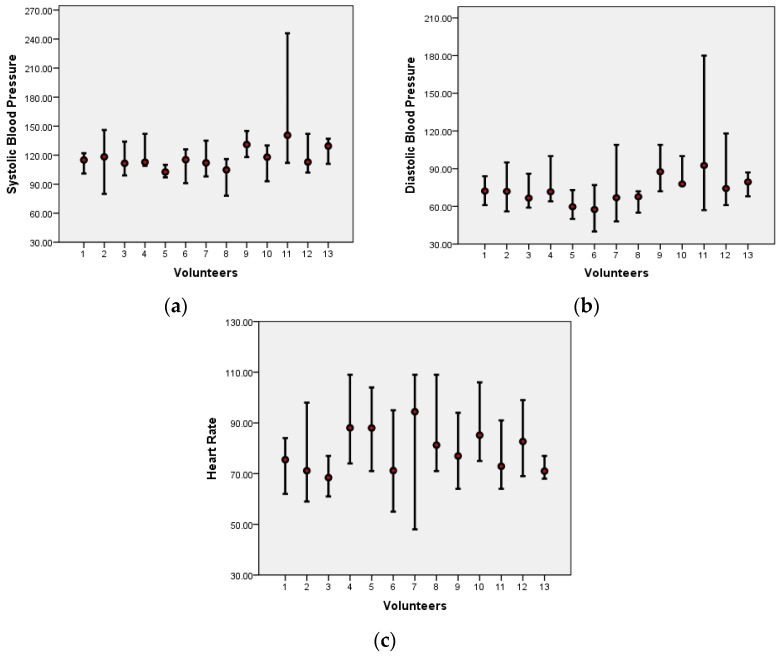
(**a**) Variation in Systolic Blood Pressure (mmHg). (**b**) Variation in Diastolic Blood Pressure (mmHg). (**c**) Heart Rate Variation (mmHg).

Table 3 summarizes the average physiological parameters, such as heart rate and blood pressure, measured every 15 min throughout the workday. The mean systolic blood pressure (SBP M) and mean diastolic blood pressure (DBP M) are generally within normal ranges for most participants, with some variations observed in a few individuals. However, blood pressure spikes above the typical values (129 mmHg × 84 mmHg), according to the new hypertension guidelines by the Brazilian Society of Cardiology [56], were recorded in 9 out of the 13 participants. Heart rate (HR) also fluctuated during telework, with values exceeding 100 bpm in 6 participants, as shown in Figure 1a–c.

Table 4 presents the results of the complete blood count (CBC) collected from each participant on one of the three days of the study, aiming to assess the blood cell lineages. Initially, we examined the erythrogram, which includes three components: red blood cells, hemoglobin, and hematocrit levels. Reference values for these components are derived from the values occurring in 95% of the population, considered as follows: Red Blood Cell count (RBC) values ranging from 4.5 to 6.0 million/mm^3^, Hemoglobin levels from 12.8 to 17.8 g/dL, and Hematocrit levels from 39 to 53%. Through the table, we observed a reduction in the number of red blood cells and hematocrit levels in some volunteers, which may suggest signs of anemia. However, hemoglobin values fall within the normal range for all participants.

The leukogram, which evaluates the levels of defense cells, represented by leukocytes and lymphocytes, with reference values of 4000–11,000/mm^3^ and 900–4000/µL, respectively, suggests that there are no infectious processes among the employees during the collection. The cortisol levels collected in blood and saliva, aimed at assessing stress levels in the body, have reference values of 6.7–22.6 mcg/dL (morning) for blood <0.736 µg/dL (morning) and <0.252 µg/dL (afternoon) for saliva. Our results show average values for most participants but with a tendency toward the lower range of the reference values.

### 3.3. Ergonomic Analysis of Workstations

Table 5 presents the physical characteristics of the home offices, arranged according to the location of the workstation in relation to natural lighting and ventilation opening. This set is based on [57], which states that the location and layout of the workstation are factors that influence occupant satisfaction through thermal and visual comfort in the workplace and improve focus and productivity [58].

Regarding the specific nature of a domestic remote working environment, where activities are carried out on a daily basis at a time predetermined by the company, certain variables are important to analyze from an ergonomic point of view, unlike other types of remote working environments, as discussed in the following sections.

### 3.4. Lighting

Various visual comfort metrics can be used to assess lighting conditions. Illuminance is a measure of the intensity of illumination on a surface and can be used as a measure of visual discomfort [59], with 500 lux being the illuminance limit considered adequate for office work environments [44,60]. Table 6 shows the average values ($\bar{X}$_L1_, $\bar{X}$_L2_, and $\bar{X}$_L3_) of illuminance measured on the horizontal plane, on the surface of the teleworkers’ desk, near the work equipment while they were carrying out their work tasks on three consecutive days.

The results of the measurements show that at no time during the teleworkers’ working day was the illuminance level within the range stipulated by the standards. At some workstations, the measured values were very low, reaching average values of 23.67 Lux (SD = 9.28), even though they were close to windows.

### 3.5. Thermal Comfort

Thermal comfort has a major impact on worker productivity in office environments [20,61]. Air temperature is a commonly used indicator to characterize the thermal environment. In this sense, NR 17 recommends that the effective temperature index should be between 20 and 23 °C in places where intellectual activities or tasks requiring constant attention are carried out [62]. Table 7 shows the average temperature values during the teleworkers’ entire working day on the three measurement days. It can be seen that in most of the work environments studied, the temperature values were above 27 °C on all three measurement days.

### 3.6. Non-Ionizing Radiation

ELF-NIR (Extremely Low-Frequency Non-Ionizing Radiations) are emitted by household devices, power transmission lines, and distribution of electrical energy, being associated with both childhood and adult cancers (such as female breast tumors, uterine cancer, brain cancer, and lymphoma), as well as reproductive problems [63,64,65]. The public exposure limit to NIR at frequencies of 50 Hz to 300 Hz is up to 200 µT, according to the International Commission on Non-Ionizing Radiation Protection [66]. However, health effects are observed with exposures starting from 0.4 µT, with risks of leukemia development in children and electrohypersensitivity in adults [22]. In this regard, our research aims to identify the level of exposure to ELF-NIR and track potential health risks for telecommuters working from home. Measurements of ELF-NIR were conducted throughout the telecommuters’ workday for 3 consecutive days.

Table 8 presents the average values of ELF-NIR. In addition to the average values, the occurrences of values above 0.4 µT were also considered.

**Figure 2:**
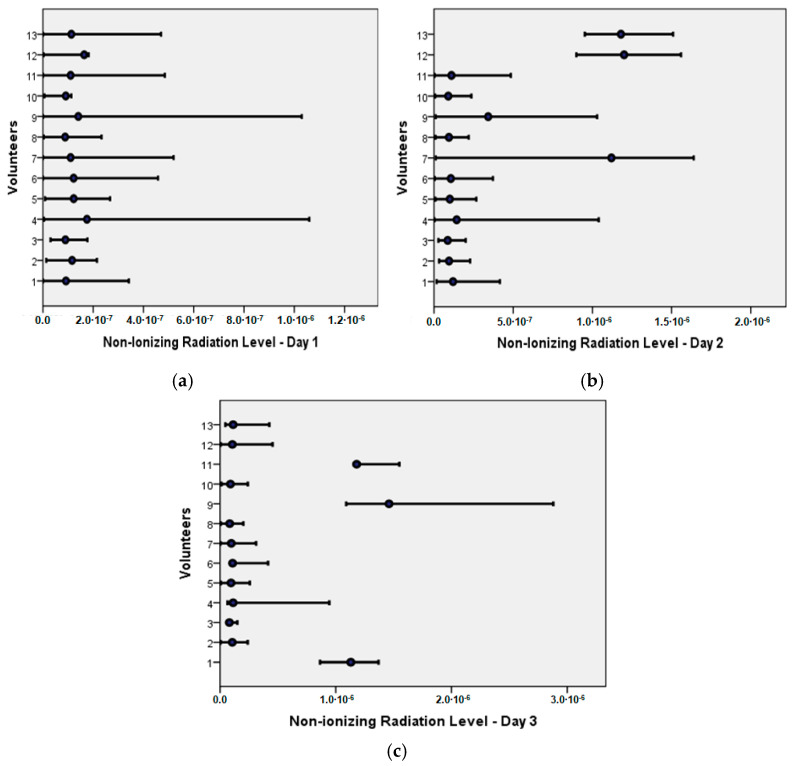
Average ELF-NIR Levels (T): (**a**) Day 1; (**b**) Day 2; (**c**) Day 3.

The average ELF-NIR exposure values were lower than 0.4 µT in most of the HO evaluated. But in some HOs, the NIR level exceeded 0.4 µT more than 200 times during the three consecutive days of measurements. And some HOs showed NIR > 0.4 µT, as shown in Figure 2.

**Figure 3:**
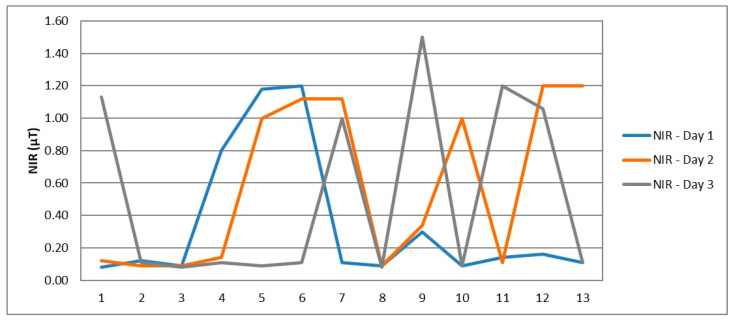
Variations in electromagnetic field levels.

Average NIR levels in telecommuting workstations vary considerably, reaching values as high as 1.5 µT >> 0.4 µT, as shown in Figure 3.

### 3.7. Professionals’ Perception of Eye Fatigue Symptoms, Lighting Conditions, and Musculoskeletal Discomfort

**Figure 4:**
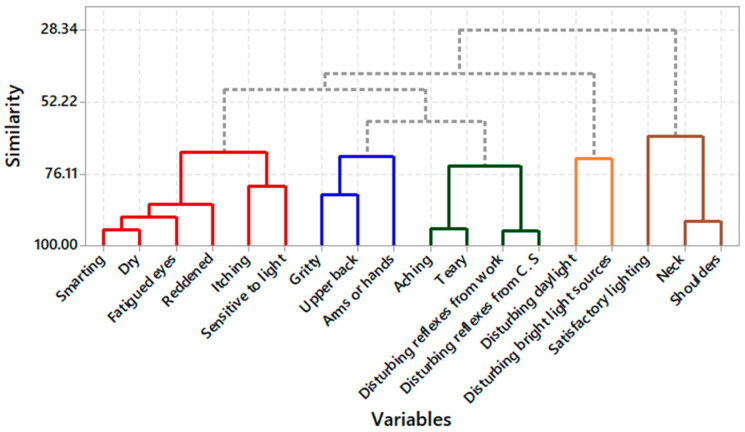
Dendrogram complete linkage; correlation coefficient distance. (Note: Different colors identify the clusters based on similarities).

The VERAM instrument obtained responses from 13 professionals regarding symptoms of eye fatigue, lighting conditions, and musculoskeletal discomfort. The results were grouped into clusters based on the degree of similarity and are represented by the dendrogram in Figure 4.

Table 9 contains eigenvalues from the correlation matrix. The eigenvalues linked to the dendrogram in Figure 4 indicate the importance of each principal component. The variance ratio indicates the relative contribution of each component, and the cumulative variance shows the importance of the five components associated with the sixteen variables mentioned in the dendrogram.

**Figure 5:**
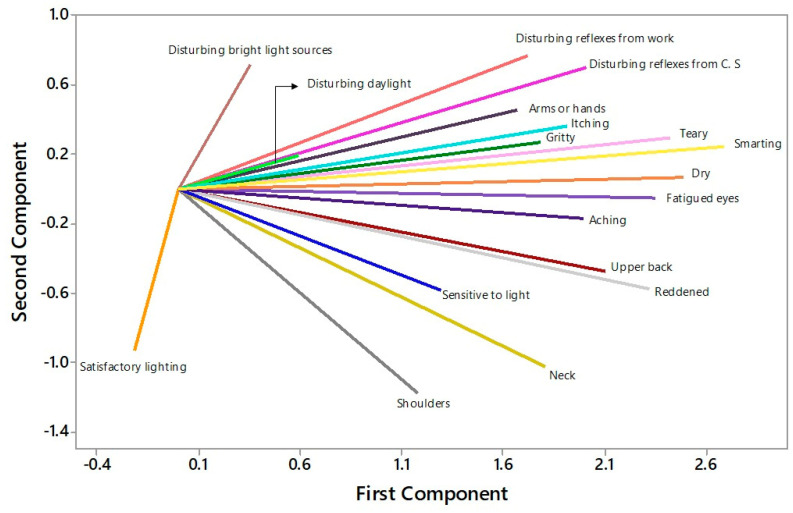
Biplot for principal components (Note: Different colors identify the original variables distributed with respect to principal components).

Figure 5 illustrates the distribution of the original variables according to the loadings of the principal components. Loadings range from −1 to 1, and the closer the variable is to these values, the greater its influence on the component.

### 3.8. Non-Ionizing Radiation and the Health of Telecommuters

Few studies have investigated the effects of NIR on human health in the extremely low-frequency range. Table 10 shows that SBP has a moderate relationship and is directly proportional to the NIR. The volunteers’ cortisol level has a moderate correlation and is inversely proportional to the NIR level.

## 4. Discussion

It should be noted that this article is not linked to any remote environment but to the Home Remote Working Environment (HRWE), specifically for professionals who work from home for 6 to 8 h a day with 1 h for lunch. This article explores the ergonomic and health impacts in this context, which has become prevalent during and after the COVID-19 pandemic. This exploratory study aims to provide an initial analysis of ergonomic and health conditions in HRWE, a topic of growing relevance in the face of changes in the global labor market.

It should be emphasized that this article offers a rich and detailed view of the professionals’ experiences, allowing for an in-depth qualitative analysis. The homogeneity of the sample in terms of the type of work and remote working conditions in the homes reduces variability, thus increasing the reliability of the results obtained. In addition, the findings of this study are in line with existing literature, reinforcing its validity. And according to [67], globalization and technological progress have recently led to teleworking arrangements from professionals’ homes, and consistent evidence on the relationship between remote working from home and ergonomic aspects and the health of professionals is scarce.

It can be seen that the lack of studies on many results is relevant and important, indicating a vast knowledge gap that is crucial to fill when determining how to implement remote home working in future working life. And this gap explains why ideal ergonomic standards for HRWE are not included in Brazilian regulatory standards, such as NR 17-Ergonomics. The quality of the data collected in this study is linked to the rigorous methodology adopted, observing and analyzing health and ergonomic aspects, including clear criteria for selecting participants, validated assessment instruments, and detailed analysis procedures. Future studies with more representative samples in HRWE can build on the results presented, and it is hoped that this article will be crucial in presenting trends and generating hypotheses that will guide subsequent research.

### 4.1. Ergonomic Analysis of Workstations

As shown in Table 5, approximately 70% of the volunteers perform their tasks in their bedrooms. This sample shares a common characteristic with recent studies in the literature, which indicate that many telecommuters conduct their professional activities in areas intended for domestic life, such as bedrooms and living/dining rooms, with nearly half of these environments lacking suitable furniture. Ergonomically speaking, these workspaces are not ideal [68]. Another study conducted during the COVID-19 pandemic found that 36% of individuals worked in bedrooms, 25% in home offices, 20% in dining rooms, and 16% in living rooms, with the bedroom considered the least conducive location for work [69].

Regarding room sizes, the usable area of these rooms ranges from 6.5 to 18.47 m^2^, with ceiling heights ranging from 2.35 to 2.65 m. Most of these home offices are oriented toward the North and South. These findings align with previous studies [70], which found home office areas ranging from 10 to 20 m^2^, ceiling heights of 3 m, and rooms with one or two windows providing a view of the sky.

NBR 15575/2021 [71] establishes requirements for functionality and accessibility in buildings. According to this standard, the minimum ceiling height should not be less than 2.50 m, a requirement met by 4 of the home offices. Concerning room orientation, facades facing south or east facilitate control of internal solar radiation [70].

The most commonly used tools in home offices were desks, chairs, laptops, mice, and external keyboards. Analyzing these tools is crucial, as poorly positioned furniture (chairs, tables), screens, keyboards, or peripherals can lead to improper posture and the onset of cervical, dorsal, lumbar, shoulder, neck, arm, wrist, and hand pain [72].

Regarding the workstation setup, most home offices had desks and chairs with dimensions described in Table 5. Only Home Office 11 conducted its tasks at the living room table. NBR 9050:2020 [73] concerning accessibility to buildings, furniture, spaces, and urban equipment, in item 4.6.3, details that the reach area of work surfaces should have a height of 0.75 m to 0.85 m from the floor to its upper surface. From this perspective, Home Offices 4, 6, 8, and 12 do not meet the requirements of this standard.

Regarding the width of the workstation, the same standard states that accessible desks or work surfaces should have a minimum free depth of 0.50 m. Observing the sample, Home Offices 1, 2, 7, and 8 do not meet the standard’s requirements.

Regulatory Standard 17—Ergonomics NR 17 [70], which establishes guidelines and requirements for adapting working conditions to workers’ psychophysiological characteristics, states that furniture should allow for postural variations, with easily adjustable settings to provide sufficient space for user comfort. The chair is one of the main components of a home office. Of the total, Home Offices 2, 8, 10, 11, and 12 do not have an ergonomic chair with backrests and adjustments. The specifications for the best chairs are adjustable height, armrests, five casters, and lumbar support on the chair back [74]. Using non-adjustable chairs, working for long periods with computers facing windows, and adopting a curved posture without support can lead to severe problems such as eye fatigue, shoulder pain, back pain, arm pain, wrist pain, and neck pain [75].

The use of laptops is widespread in workspaces. Of the total volunteers, only Home Offices 4 and 9 use desktop computers; in the case of Home Office 9, it also uses a second external monitor. The remaining volunteers use laptops. Home Office 8 uses two laptops. Recent findings on ergonomics and discomfort in home offices have shown that approximately 85% of telecommuters use laptops, and only 45% have an external monitor [74]. The exclusive use of laptops by many workers, with screens positioned very low, combined with inadequate chairs without lumbar support, stands out as one of the main problems with home offices [76].

The addition of peripheral devices to laptops was performed by approximately 92% of the volunteers. Home Offices 1 to 6 and 9 had external mice and keyboards; Home Offices 7 and 10 used only the mouse, and Home Offices 8 and 11 did not use any peripheral devices. Literature mentions that the use of the laptop keyboard and mouse is directly related to discomfort in the forearm, elbow, and arm [66]. In order to keep the elbow and wrists in a neutral position during use and minimize shoulder movement, it is recommended to position the external mouse next to the external keyboard and on the same surface as the latter [77].

Regarding shading devices, about 50% of the Telework Stations contain this component, either translucent or blackout curtains. Like the facade orientation and room size, the use of shading devices is considered a component capable of influencing user satisfaction, as it allows for regulating natural lighting [70].

### 4.2. Lighting

The comfort of occupants in the home office is associated with environmental variables such as lighting, as these influence work concentration and productivity [78]. Lighting conditions that can affect the performance and well-being of workers include the brightness of screens, natural light, and lighting levels at the workstation [79]. If appropriate, lighting in the home office promotes comfort and satisfactory productivity [80]. Otherwise, poor lighting can trigger illnesses [81] such as fatigue, tearing, and redness [29].

According to Table 6, HO 8 and HO 6 exhibited the lowest and highest lighting levels, respectively, although all values of the HOs are below the recommended standard. Most HOs located perpendicular to natural lighting and ventilation openings showed more satisfactory values compared to those positioned perpendicular to lighting and ventilation openings. Placing the workstation on the sides of the user avoids possible light reflections that affect the visual satisfaction of an individual using a computer screen [66].

The main objective of lighting is to provide support for the environment to have adequate visual conditions for carrying out activities with comfort, precision, speed, and safety [82]. In Brazil, Ref. [44] deals with lighting in work environments and establishes minimum requirements for people to perform their visual tasks efficiently, comfortably, and safely during working hours. According to this standard, for working with VDT (Video Display Terminal), computer-assisted offices, and activities involving reading, writing, typing, and data processing, the recommended lighting is 500 lux.

### 4.3. Temperature

The assessment of air temperature in the home office is essential to ensure thermal comfort, which can affect workers’ performance [83], and it interferes with the optimization of energy use [84]. When workers feel neutral or slightly cold, they are more likely to reach a state of optimal productivity [85].

By analyzing the air temperature, it can be observed that the values in the HOs range between 25 °C and 31 °C. The minimum value, referring to HO 6, shows a high standard deviation caused by the activation of the air conditioning device during the measurement. HOs marked with temperatures above 30 °C are positioned to the North, which leads to the belief that the room’s solar orientation influenced the results since the northern face receives the most sunlight [86].

ISO 7730/2005 [87] suggests that thermal comfort should achieve a temperature of 23 °C. The literature recommends the ideal temperature range for office thermal comfort is 21 to 25 °C [88]. Studies that have developed artificial neural network (ANN) models for predicting thermal comfort in indoor environments through thermal sensations and occupant behavior mentioned that the acceptable air temperature range for offices is 20.6 °C (69 °F) to 25 °C (77 °F) in winter and 20.6 °C (69 °F) to 25.6 °C (78 °F) in summer [63]. Thermal comfort in offices is achieved by maintaining the temperature between 20 °C and 25 °C and is influenced by airflow and temperature within the workspace [89].

### 4.4. Non-Ionizing Radiation

Aware that remote home office work environments are characterized by the use of information and communication technologies [90], levels of non-ionizing radiation (NIR) were investigated. Brazilian legislation regarding NIR [91] does not establish parameters on exposure limits, only mentioning that operations or activities that subject workers to black light radiation (ultraviolet in the range 400–320 nanometers) will not be considered hazardous.

The Law Nº 11.934/2009 [92] addresses human exposure limits to electric, magnetic, and electromagnetic fields associated with the operation of radiocommunication transmitters, user terminals, and electrical power systems in frequency bands up to 300 GHz (three hundred gigahertz), aiming to guarantee the protection of health and the environment.

As described in the law, it is the responsibility of ANEEL to regulate and monitor compliance with the recommended electric and magnetic field exposure limits by WHO. The limits refer to the general public’s and the occupational population’s exposure to electric and magnetic fields. According to [93], for a frequency of 60 Hz, the reference levels established by ICNIRP and recommended by WHO for the time-varying electric and magnetic fields for the general public are 4.17 (kV/m) and 200 µT, respectively, whereas, for the occupational population, it is 8.33 (kV/m) and 1000 µT.

However, these parameters refer to exposure limits to electric and magnetic fields originating from electric power generation, transmission, and distribution installations, and it is known that in the home office environment, there are devices that go beyond these specifications.

Regarding adverse health effects, scientific evidence suggests that they depend on factors such as exposure period, radiation intensity, frequency, or type of radiation [94]. Some individuals show sensitivity to electromagnetic fields and develop dermatological symptoms such as dizziness, nausea, tingling, and difficulty concentrating [94]. Other studies mention that the electromagnetic field does not interfere with the functioning of living organisms as long as acceptable standards are respected [95].

Furthermore, scientific evidence describes that technological advancement has a cost to human and environmental health, and this price has already begun to be paid, with a tendency to increase as exposure time increases. As actions to minimize exposure to electromagnetic pollution are postponed, the cost in terms of public health, quality of life, work absenteeism, and increased morbidity will be higher [62].

### 4.5. Perception of Professionals Regarding Symptoms of Eye Fatigue, Lighting Conditions, and Musculoskeletal Discomfort

Symptoms such as burning sensation, dryness, tired eyes, redness, itching, and photophobia (light sensitivity) showed 68.95% similarity, grouped in the red cluster, and could be considered the same variable. The same occurred with symptoms of eye, back, hand, or arm pain, which showed 70.43% similarity, grouped in the blue cluster; sensation of grittiness in the eyes, tearing, discomfort with reflections on the work surface, and discomfort with computer screen reflections showed 73.48% similarity in the green cluster; satisfactory lighting at work, neck, and shoulder pain showed 63.70% similarity in the brown cluster. The clustering of these clusters is consistent with the literature, as dry eyes, burning sensation, light sensitivity (photophobia), and neck or shoulder pain are symptoms of computer vision syndrome [96,97].

By performing principal component analysis, it was observed that five principal components are sufficient to explain more than 80% of the original variables’ variability, reducing the problem’s dimensionality and revealing the correlation structure of ergonomic aspects. The first five components consist of combined loads of the original analyzed variables. The spreading of loads from the original variables in the two-dimensional plane shows that the symptom of burning sensation has strong positive correlations with tearing and dryness and a negative association with satisfactory lighting. Furthermore, it is observed in Figure 5 that discomfort in the shoulder and neck has positive loads with the first component and negative loads with the second component. This result agrees with findings from the literature, which show an association between visual, ocular, and musculoskeletal symptoms in the neck and shoulder [98,99,100,101].

### 4.6. Relationships between NIR and the Health of Home Office Teleworkers

One of the objectives of this study was to verify the levels of public exposure to extremely low-frequency magnetic fields in HO workplaces and track possible repercussions of the high rates on the health of teleworkers. According to the data obtained, we can verify that some HOs had higher levels of ELF-NIR than others, even using similar devices such as notebooks, cell phones, computer monitors, and tablets. The observed differences may be associated with the greater number of electrical appliances in the home environment or the greater proximity of the workstation to internal electrical wiring points. According to [102], proximity to household appliances can increase NIR in residential environments. Furthermore, residential exposure to electromagnetic fields may be related not only to internal sources but also to external sources. The proximity of homes to elements such as transformers, high voltage lines, and even electrical substations can add electromagnetic field intensity, also influencing internal measurements [103,104].

The values measured in this research, close to the HO station, varied between maximum values of 1.5 µT (SD = 0.31) and minimum of 0,07 µT (SD = 0.02), with averages between 1.4 µT and 0.08 µT. The study [19], which evaluated exposure to ELF-NIR in residential environments, obtained values that varied between 0.187 µT and 0.09 µT in bedrooms and 0.08 µT and 0.09 µT in living rooms, being higher when close to household appliances. The study by [105], also carried out in residential environments, obtained values ranging from 0.12 µT to 0.49 µT. For [74], exposure levels of up to 200 µT are accepted, also considering the exposure time and field frequency. For [21], exposure to magnetic fields above 0.4 μT is sufficient to cause biological changes in children and older adults.

The group studied was HO teleworkers, with daily working time between 6 and 8 h, 5 days a week, 1 h for lunch, living in buildings in neighborhoods considered heat islands, in the city of João Pessoa—PB. The group’s lifestyle and way of working are quite similar, with the majority being overweight (BMI ≥ 25 to 29.9 kg/m^2^—overweight), but with waist measurements outside the values considered to be at risk for developing cardiovascular diseases (≥102 cm in men and ≥88 cm in women) [106]. They present sedentary behavior, with little physical activity during the week, especially during teleworking, which can lead to aches, higher levels of stress, obesity, and diseases such as high blood pressure. According to [107], teleworking has little impact on physical activity. However, in the face-to-face work model, social support contributes to participation in health activities at work.

In responses to questions involving health included in the sociodemographic questionnaire, the majority of teleworkers consider themselves to be in good health despite occasional complaints of body pain, especially head and eye pain. In a review on the topic, Ref. [12] identified that home teleworkers complained of migraines, eye strain, shoulder tendonitis, back pain, neck pain, and wrist pain; still, they consider these symptoms to be trivial, in compared to the stress they experienced in their offices.

Regarding physiological parameters, the blood pressure values of teleworkers presented normal average values. Nevertheless, peak values of both systolic and diastolic blood pressure were observed, above expected values, in several measurements during the day in most volunteers. In the case of blood pressure, its variability is recognized as a potential risk factor for cardiovascular diseases and a predictor of stroke and coronary events in high-risk patients [108]. It can be affected by many factors, such as behavioral, emotional, and postural [109]. The relationship between blood pressure and NIR-EXF levels identified in this study, with correlations for BPS M (0.514, *p* = 0.035), MAX PAS (0.587, *p* = 0.021), and SBP MIN (0.486, *p* = 0.048), is not very clear and could be considered an adverse health effect depending on the outcome on the population studied [110].

According to Table 8, the inversely proportional relationship observed suggests that, within the domestic remote working environment, there is an association between higher NIR exposure and lower cortisol levels (R = −0.622, *p* = 0.023 < 0.05). Cortisol is a steroid hormone responsible for acting on several physiological processes in the human body in stressful situations. According to [111], exposure to NIR induces cellular stress triggered by cellular macromolecular damage to proteins, lipids, and DNA to repair and return cellular functions to homeostasis, which can alter cortisol levels. Low cortisol levels can cause symptoms of depression, tiredness, cravings for sweets, and weakness, or even trigger Addison’s disease, a disorder in which the adrenal glands do not produce enough hormones.

## 5. Conclusions

In this study, home office (HO) workstations in high-rise residential buildings in the heat island regions of João Pessoa were evaluated from an ergonomic point of view. It was found that in terms of furniture, heat, and light, none of the HOs were ergonomically suitable, demonstrating the lack of guidance and support for the proper implementation and configuration of the workstation. Some of these inadequacies may be linked to symptoms such as burning sensation, dryness, tired eyes, redness, itching, and photophobia (sensitivity to light) mentioned by the volunteers.

The air temperature was higher than 25 °C in the home remote working environments studied, which is not in line with thermal comfort recommendations and ergonomic principles when it comes to intellectual activities, although there is no specific regulatory standard that determines a maximum temperature value for home remote working environments in Brazil.

The illumination levels at the workstations are well below those recommended by the standard, even in offices situated on the side of the window, which should have led to better lighting levels. The visual and musculoskeletal discomfort reported by the teleworkers reflects such inadequacy.

The electromagnetic field measurements of some HOs were above (0.4 µT), signaling a warning regarding the risk posed by extremely low-frequency electromagnetic pollution. Prolonged exposure to non-ionizing radiation (NIR) can affect teleworkers’ health; in this regard, parameters such as blood pressure and cortisol levels appear to be more sensitive to variations in NIR.

### Limitations of This Study

A limitation of this study was the less-than-ideal adherence of volunteers, even with the use of a convenience sample. This is because the participants were reluctant to be evaluated from a health point of view, in addition to the logistical difficulties associated with recruiting professionals. To mitigate this impact, several additional recruitment attempts and methodological adjustments were made. Although this limitation may have influenced the representativeness of the data, it is essential to consider that the environment analyzed is specific and unique, being a domestic remote working environment. This specificity offers valuable information on the ergonomic and productivity impacts in this particular context. The results obtained provide significant and unprecedented data on the subject in question and could serve as a basis for future research with larger and more diverse samples.

## Figures and Tables

**Table 1:** Sociodemographic characteristics.

VOL	Sex	Age	Marital Status	Children	Educational Level	Profession	Company Type	Time of HO	Working Time
Vol1	M	48	Married	None	Superior	Professor	Public	3 years	8 h
Vol2	F	36	Married	None	Superior	Engineer	Private	5 years	8 h
Vol3	M	37	Married	None	Superior	Professor	Public	15 years	8 h
Vol4	M	32	Married	1	Superior	Engineer	Public	3 years	8 h
Vol5	F	28	Single	None	Superior	Engineer	Public	18 months	8 h
Vol6	M	33	Married	None	Superior	Engineer	Public	1 year	8 h
Vol7	F	35	Married	1	Superior	Engineer	Private	2 years	8.5 h
Vol8	F	47	Married	2	Superior	Engineer	Private	10 years	6 h
Vol9	F	48	Married	1	Superior	Therapist	Autonomous	1 year	8 h
Vol10	M	36	Married	None	Superior	Professor	Public	15 years	6 h
Vol11	F	70	Married	1	Medium	Admin Technician	Private	49 years	6 h
Vol12	F	36	Married	None	Superior	Psychologist	Autonomous	10 years	6 h
Vol13	M	58	Married	1	Superior	Admin Technician	Public	39 years	7 h

**Table 2:** Health profile.

VOL	BMI (Kg/m^2^)	Waist (cm)	Smoker	Drink	Declared Physical Activity	Number of Steps Measured	Health Perception	Health Complaint
Vol1	24.2	80	N	Weekends	Always	2200	Good	None
Vol2	25.5	89	N	Almost never	Weekends	345	Good	Body pain and anxiety
Vol3	29.3	91	N	Weekends	Always	241	Good	None
Vol4	22.4	75	N	Almost never	Weekends	1700	Good	Poor blood circulation
Vol5	21.7	74	N	Weekends	Always	550	Good	Reflux, pain in the eyes and head
Vol6	26.31	90	N	Almost never	Weekends	350	Good	None
Vol7	27.09	88	N	Almost never	Always	194	Good	None
Vol8	26.49	67	N	Nunca	Weekends	900	Good	Forgetfulness
Vol9	29.16	79	N	Almost never	Almost never	1500	Good	Pain in the eyes
Vol10	19.13	68	N	Almost never	Almost never	700	Good	Pain in the eyes
Vol11	26.8	89	N	Weekends	Weekends	289	Good	Body aches
Vol12	23.39	62	N	Weekends	Weekends	4000	Good	Poor blood circulation
Vol13	28.36	92	N	Weekends	Weekends	300	Good	Joint pain (hands and neck)

**Table 3:** Physiological parameters.

VOL	SBP M (mmHg)	DBP M (mmHg)	HR M (BPM)
Vol1	111 ± 3.9	72.2 ± 3.6	75.5 ± 7.5
Vol2	118.6 ± 12	71.9 ± 8.37	71.1 ± 0.5
Vol3	111.58 ± 6.20	66.61 ± 5.86	68.4 ± 3.2
Vol4	112.7 ± 3	71.63 ± 4.76	88 ± 6.5
Vol5	102.6 ± 4	59.71 ± 4.00	88 ± 4.9
Vol6	115.2 ± 7	57.56 ± 6.98	72 ± 6.3
Vol7	112.03 ± 7	66.87 ± 6.95	94 ± 4.4
Vol8	104.76 ± 4	67.67 ± 2.40	81 ± 5.3
Vol9	130.97 ± 7	87.59 ± 6.25	76 ± 5.5
Vol10	117.9 ± 5	77.8 ± 5.28	85 ± 5.5
Vol11	140 ± 24	92.55 ± 0.89	72 ± 5.0
Vol12	112 ± 8	74.2 ± 8.42	83 ± 7.2
Vol13	129.42 ± 6	79.65 ± 3.66	72 ± 3.0

**Table 4:** Blood count and cortisol levels.

VOL	N° RBC (millions/m^3^)	HOG g/dL	HET (%)	LEC (/mm^3^)	LIF (/mm^3^)	COR BLOOD (mcg/dL)	COR MOR (µg/dL)	COR AFT (µg/dL)
Vol1	4.84	14.3	42.4	6.300	1.651	10.1	0.38	0.2
Vol2	4.7	13.2	39.6	7.300	2.132	12.6	0.11	<0.054
Vol3	5	15	43.9	6.900	3.008	15.4	0.27	0.13
Vol4	4.87	14.1	41	4.900	1.730	4.6	0.054	<0.054
Vol5	4.3	13.4	39.7	7.900	2.899	11.38	0.32	<0.054
Vol6	5.8	14.3	44	6.200	2.201	8.9	0.16	<0.054
Vol7	3.9	12	35.7	7.600	2.622	7.03	0.11	<0.054
Vol8	3.9	12.1	34.2	5.400	1.717	9.89	0.18	<0.054
Vol9	4.4	12.9	3.4	10.000	3.700	14.90	0.28	<0.054
Vol10	5.2	14.9	43.5	8.200	3.059	13.7	0.36	<0.15
Vol11	4.17	12.9	37.4	4.100	1.222	5.89	0.23	<0.054
Vol12	3.91	12.9	38.5	4.500	1.251	10.1	0.34	<0.054
Vol13	4.68	12.8	39.6	4.100	968	6.01	0.16	<0.07

Main CBC data: N° RBC—Red blood cells; HOG—Hemoglobin; HET—Hematrocrits; LEC—Leukocytes; LIF—Lymphocytes; COR BLOOD—Cortisol in the blood; and COR MOR—Cortisol saliva morning and COR AFT—Cortisol saliva afternoon.

**Table 5:** Home office characteristics.

**Location—Perpendicular to Lighting and Ventilation Openings**								
**HO**	**Room**	**Area** **(m^2^)**	**Ceiling High (m)**	**Room Orientation**	**Workstation Depth (cm)**	**Workstation Height (cm)**	**Ergonomic Chair with Adjustments and Rounded Edge**	**Shading Devices**
1	Bedroom	10.0	2.45	North	45	77	Yes	Yes
2	Bedroom	12.0	2.45	North	45	75	No	Yes
4	Home office	6.56	2.55	East	60	73	Yes	No
5	Bedroom	7.48	2.62	East	52	75	Yes	Yes
6	Home office	7.17	2.44	West	60	120	Yes	Yes
8	Bedroom	6.80	2.45	North	35	87	No	No
11	Living room	11.19	2.50	North	80	76	No	No
12	Bedroom	18.47	2.65	North	55	89	No	Yes
13	Bedroom	9.40	2.51	North	45	120	Yes	Yes
**Location—Parallel to Lighting and Ventilation Opening**								
**HO**	**Room**	**Area** **(m^2^)**	**Ceiling High (m)**	**Room Orientation**	**Workstation Depth (cm)**	**Workstation Height (cm)**	**Ergonomic Chair with Adjustments and Rounded Edge**	**Shading Devices**
3	Bedroom	8.84	2.35	South	60	79	Yes	Yes
7	Bedroom	7.05	2.45	South	45	75	Yes	No
9	Bedroom	8.31	2.27	West	60	75	Yes	No
10	Bedroom	7.94	2.53	South	55	75	No	Yes

**Table 6:** Lighting.

HO	$\bar{X}$_L1_ (lux)	SD	$\bar{X}$_L2_ (lux)	SD	$\bar{X}$_L3_ (lux)	SD
1	167.24	95.59	360.63	53.42	217.37	107.72
2	109.14	32.03	111.05	26.30	90.41	44.97
3	54.03	16.94	70.70	19.62	62.47	18.10
4	218.90	87.45	228.72	81.62	222.47	85.84
5	181.85	146.49	309.55	112.39	201.96	96.91
6	290.91	171.02	138.85	161.52	259	171.92
7	90.41	44.97	85.52	54.08	62.35	63.02
8	23.67	9.28	25.69	11.09	23.9	9.51
9	226.38	122.17	228.06	84.70	225.19	63.53
10	27.98	11.20	27.85	17.86	27.90	15.14
11	34.25	45.61	35.74	20.78	35.74	20.78
12	93.17	19.71	92.82	18.93	97.54	15.89
13	105.58	50.34	128.48	49.71	98.31	47.97

**Table 7:** Temperature.

HO	$\bar{X}$_T1_ (°C)	SD	$\bar{X}$_T2_ (°C)	SD	$\bar{X}$_T3_ (°C)	SD
1	29.80	0.28	30.80	0.51	31.00	0.52
2	27.1	0.50	29.1	0.64	28.2	0.24
3	27.40	0.29	27.50	0.33	27.90	0.13
4	29.50	0.26	28.60	0.23	28.20	0.38
5	28.30	0.35	28.30	0.34	28.50	0.43
6	25.40	1.98	25.70	2.40	25.00	2.12
7	29.00	0.23	29.40	0.24	28.60	0.17
8	27.70	0.14	28.60	0.27	27.80	0.14
9	28.90	0	28.40	0.33	29.30	0.05
10	29.40	0.21	29.20	0.18	29.50	0.20
11	30.50	0.15	28.80	0.37	28.50	1.02
12	30.00	0.18	26.60	1.26	29.90	0.22
13	29.59	0.72	30.37	0.73	30.15	0.66

**Table 8:** Level of ELF-NIR in home office environments.

HO	$\bar{X}$_1_ (µT)	SD (µT)	Case >0.4 µT	$\bar{X}$_2_ (µT)	SD (µT)	Case >0.4 µT	$\bar{X}$_3_ (µT)	SD (µT)	Case >0.4 µT
1	0.09	0.05	0	0.12	0.05	0	1.12	0.05	83,556
2	0.11	0.03	0	0.09	0.09	0	0.11	0.06	0
3	0.07	0.02	0	0.08	0.02	0	0.08	0.02	0
4	0.17	0.09	4004	0.14	0.07	1511	0.11	0.05	246
5	1.17	0.06	3339	0.10	0.04	0	0.09	0.04	0
6	0.12	0.07	84	0.10	0.06	0	0.11	0.07	2
7	0.11	0.06	92	1.12	1.57	27,972	0.10	0.06	0
8	0.08	0.03	0	0.09	0.03	0	0.08	0.03	0
9	0.24	0.14	3832	0.34	0.02	1899	1.5	0.31	4920
10	0.09	0.03	0	0.09	0.03	0	0.09	0.03	0
11	0.14	0.08	153	0.10	0.06	23	1.12	0.07	4920
12	0.16	0.12	4892	1.20	0.09	32,227	0.11	0.08	6
13	0.11	0.06	11	1.18	0.05	7025	0.11	0.06	2

**Table 9:** Eigenanalysis of the correlation matrix.

Component	1	2	3	4	5	6	7	8	9	10
Eigenvalue	7.8809	2.5398	1.9094	1.6153	1.3837	0.9679	0.6969	0.4784	0.2463	0.1371
Proportion	0.438	0.141	0.106	0.090	0.077	0.054	0.039	0.027	0.014	0.008
Cumulative	0.438	0.579	0.685	0.775	0.852	0.905	0.944	0.971	0.984	0.992

**Table 10:** Relationship between physiological and biochemical parameters and ELF-NIR.

Spearman’s Correlation	NIR M	NIR MAX	NIR MIN
SBP M	R = 0.514; *p* = 0.035 < 0.05	R = 0.587 *p* = 0.021< 0.05	R = 0.486 *p* = 0.048 < 0.05
SBP M	R = 0.620; *p* = 0.024 < 0.05		
MORNING CORTISOL		R = − 0.622 *p* = 0.026 < 0.05	

## Data Availability

The data are not available for ethical reasons.
